# Sex‐specific relation of replacing sedentary time with physical activity on stage 2 hypertension incidence in middle‐aged adults

**DOI:** 10.14814/phy2.70830

**Published:** 2026-04-15

**Authors:** Sophie E. Rayner, Jolaine Katryne Bossé, Corinne Brideau, Jolaine Comeau, Jessie Drouin, Jalila Jbilou, Myles W. O'Brien

**Affiliations:** ^1^ Department of Medicine Université de Sherbrooke Sherbrooke Quebec Canada; ^2^ Centre de Formation Médicale du Nouveau‐Brunswick Université de Sherbrooke & Université de Moncton Moncton New Brunswick Canada; ^3^ School of Physiotherapy (Faculty of Health) Dalhousie University Halifax Nova Scotia Canada; ^4^ School of Psychology Université de Moncton Moncton New Brunswick Canada

**Keywords:** cardiometabolic health, cohort study, device measured activity, isotemporal substitution, sex differences

## Abstract

Hypertension is increasingly prevalent among middle‐aged adults, but the impacts of physical activity and postures are not fully understood in middle‐aged males and females, limiting targeted prevention. This study investigated associations between habitual physical activity and postures with hypertension, and whether they differ between sexes at the same age. 4416 participants (age: 46 years) in the 10th sweep of the 1970 British Cohort Study were used. Participants wore an activPAL to measure physical behaviors. Stage 2 hypertension was defined as >140/90 mmHg or antihypertensive medication use. Isotemporal substitution models assessed the theoretical effect of reallocating 30 min of one behavior with another. Males had higher mean body mass index (BMI: 28.5 ± 4.6 kg/m^2^ vs. 27.9 ± 6.0), systolic (129.1 ± 13.3 mmHg vs. 119.6 ± 14.7 mmHg) and diastolic blood pressure (79.0 ± 10.4 vs. 74.8 ± 10.7 mmHg) compared to females (all, *p* < 0.001). In pooled analyses, substituting sedentary time with light physical activity (LPA) (OR = 0.93 [0.86, 0.998]) or moderate‐to‐vigorous physical activity (MVPA) (OR = 0.90 [0.83, 0.97]) was associated with lower odds of hypertension. In males, replacing sedentary time or standing with any activity reduced hypertension risk (ORs 0.86–0.88), whereas in females, only replacing sedentary time with standing was associated with lower risk (OR = 1.04 [1.01, 1.07]). MVPA demonstrated stronger associations than LPA. Replacing sedentary or standing behaviors with movement, particularly MVPA, may reduce hypertension risk in middle‐aged adults, with more pronounced benefits observed in males. These findings support the development of sex‐specific interventions targeting physical activity behavior patterns to mitigate hypertension risk during midlife.

## 1 INTRODUCTION

Hypertension is among the most prevalent chronic conditions globally, affecting approximately one‐in‐four adults and contributing to over 10% of all‐cause mortality worldwide. Its prevalence increases with age, affecting more than 40% of adults over the age of 25 (Amagasa et al., 2018; O'Brien et al., 2018). Sex differences in the prevalence of hypertension are variable, but males tend to have higher rates of hypertension than females in middle‐age years, but this sex difference is attenuated with age (O'Brien & Theou, 2024; Olivera & Graham, 2022). A more comprehensive understanding of the role of modifiable lifestyle behaviors in mitigating hypertension risk among males and females during middle‐age years, a critical prodromal phase in the pathogenesis of hypertension, is needed.

Engaging in regular physical activity during adulthood is beneficial for blood pressure regulation, and sustained adherence to an active lifestyle over the years provides cumulative health benefits (Pouliou et al., 2012). Emerging evidence indicates the negative impacts of sedentary postures, the body position that individuals spend most waking hours, independent of physical activity (O'Brien et al., 2024). A randomized clinical trial demonstrated that reducing sitting time by 30 min/day over 6‐months reduced systolic blood pressure by ~3 mmHg in older adults (Rosenberg et al., 2024). Similarly, a large‐scale, population‐based data analysis demonstrated that higher step counts and longer durations of vigorous physical activity were associated with lower blood pressure, while prolonged sedentary time was linked to higher blood pressure (Kinuta et al., 2025). Notably, the associations were more pronounced in males under the age of 60, suggesting a possible sex‐ and age‐specific sensitivity to physical behaviors (Kinuta et al., 2025). Furthermore, these reports do not consider the co‐dependent nature of habitual activity, in that increased time spent in one activity displaces another (e.g., more physical activity time may displace sedentary time). Isotemporal substitution modeling examines the theoretical impact of replacing time spent in one behavior versus another, within the parameters of a finite day (Grgic et al., 2018). The relation between physical behaviors, such as time spent sitting or standing, and sedentary patterns and cardiovascular outcomes between sexes are not fully understood (Bosomworth & Khan, 2023; Rosenberg et al., 2024).

The 1970 British Cohort Study (1970BCS) is an ongoing longitudinal study of thousands of individuals born in the same week of April 1970 in England, Scotland, and Wales (Sullivan et al., 2023). The nature of the 1970BCS permits novel insights into aging, as participants share the same chronological age. Accordingly, this cohort is primed to answer research questions relative to chronic disease development during middle age. The sex‐specific effects regarding the impact of movement on blood pressure observed in previous studies have been inconsistent and generally small in magnitude (Bosomworth & Khan, 2023; Kinuta et al., 2025; Ramirez & Sullivan, 2018). To address this gap, our study aims to examine how device‐measured physical behaviors, including standing, stepping, and sedentary patterns, are associated with hypertension, and whether these associations differ between males and females of the same chronological age. Specifically, this cross‐sectional study using the 1970BCS determined the relation between replacing time in physical behaviors on hypertension among middle‐aged males and females. We hypothesized that (1) replacing sedentary time with activity would be associated with a lower incidence of hypertension, and (2) middle‐aged males would exhibit a stronger association between physical behaviors and hypertension than females.

## 2 MATERIALS AND METHODS

### 2.1 1970 British cohort study

Data were taken from the 1970BCS, an ongoing longitudinal cohort study consisting of individuals born in a single week of 1970 in the United Kingdom. Individuals in the cohort are currently 55 years old and have been contacted for 10 follow‐up data collections. This cross‐sectional study analyzes data from the 10th and most comprehensive sweep of the 1970BCS, collected at 46 years old, containing bio measures, objective, and subjective measures of health. No participants were excluded based on disease status, and participants were included if necessary data were available. Ethical approval for each wave of data collection in the 1970BCS was obtained by the Centre for Longitudinal Studies from the appropriate ethics committees. The present secondary analysis of de‐identified data was conducted in accordance with the terms of use of the UK Data Service. Data used for the current study are available online at https://beta.ukdataservice.ac.uk/datacatalogue/studies/study?id=8547.

### 2.2 Anthropometrics and cardiovascular measures

Stature (cm) was measured using a Leicester stadiometer, and body mass (kg) was assessed using Tanita BF‐522 W scales (Tanita Europe B.V.). Body Mass Index (BMI) was subsequently calculated using nurse‐measured values as body mass (kg) / Stature (m)^2^. Participants were asked to report their prescribed medications; nurses verified medication packaging to increase accuracy. Medications were coded by their national health service (NHS) categories. Brachial blood pressure and heart rate were measured after a five‐minute rest using an Omron HEM 907 device, with three readings taken at one‐minute intervals (Deng et al., 2024), the average of all measurements used to determine systolic (SBP) and diastolic (DBP) blood pressure (mmHg). Participants were categorized based on their blood pressure values, where individuals were defined as Stage 2 hypertensive if they had any of: SBP >140 mmHg, DBP >90 mmHg (Unger et al., 2020), or were currently on medication for managing blood pressure (nurse validated). The definition of hypertension as >140/>90 mmHg, now termed Stage 2 hypertension by the American Heart Association (Writing Committee Members*, 2025) was selected to match historical considerations of hypertension, while appreciating that this definition varies by organization and measurement type.

### 2.3 Device measured physical activity and posture

Participants that consented were fitted with ActivPAL devices (activPAL3 micro, PAL Technologies Ltd. Glasgow, UK) to objectively measure physical activity and sedentary time. The activPAL can determine thigh position and acceleration to classify time spent sitting/lying, standing upright, and stepping. The activPAL is a highly valid measure of habitual posture and physical activity (Grant et al., 2006, 2008). The activPAL was secured on the right mid‐thigh using a waterproof medical dressing by trained nurses. Participants were instructed to wear the activPAL monitor continuously for 7 days, including during sleep, bathing, swimming, and all physical activities. In addition to the device, participants completed a self‐report diary. This diary recorded the time they went to bed, the time they woke up, and the number of times they got up at night. It also captured any instances where the device was removed early. Participants were excluded if they were allergic to plasters or adhesives or to low‐density polyethylene (plastic packaging the activity monitors were sealed in), had a skin condition (e.g. broken skin, eczema on their legs), or if they were going through a metal detector/security checkpoint in the next week. Out of the 8581 participants who took part in the Age 46 Survey, 6492 consented to wearing the activity monitor. Among those, 5569 returned their monitors with at least one full day of usable data. All other participants (*n* = 4416) were included if they recorded at least 4 consecutive days of wear with at least 10 h of valid wear time per day.

Data from the ActivPALs were downloaded as DATX files and processed through activPAL3 software. The open‐access program, Processing PAL, was used to process the data (https://github.com/UOL‐COLS/ProcessingPAL/releases/tag/V1.0), which uses an algorithm to isolate valid waking wear data from sleep or prolonged non‐wear (Winkler et al., 2016) with days defined from midnight to midnight. Monitors were not replaced if removed from the thigh during collection; thus, sleeping hours were defined as 24 h minus waking hours. The program outputted total sampling averages for a wide range of outputs including sedentary time, prolonged uninterrupted sedentary time (>1 h), total standing time, step counts, stepping time, LPA, and MVPA (defined as stepping cadence >100 steps/min) (Tudor‐Locke et al., 2019).

### 2.4 Statistical analyses

All statistics were completed in RStudio (Posit Software, PBC V 2024.12.0 + 467), and significance set at *α* = 0.05. Descriptive data are presented as means ± SD or proportions (%), while logistic regressions are presented as (odds ratio (OR) [95% confidence interval] *p*‐value). Mann–Whitney *U* tests were used to compare demographics, anthropometrics, and cardiovascular measures between sexes.

Isotemporal substitution modeling was completed and examined the association of substituting sedentary time with standing time, LPA or MVPA, and LPA with MVPA on hypertension. A 30‐min daily substitution time was chosen as an achievable lifestyle modification for adults, which is the most commonly used duration in isotemporal substitution analyses (Grgic et al., 2018). Each physical activity behavior was converted into standardized units of time before being entered into the models (e.g., 30‐min analyses, ~20 units [~600 min/day ÷ 30]). Odds ratios were determined for hypertension risk (binary as 0 = non‐hypertensive, and 1 = hypertensive). Covariates included sex and body mass index (BMI).

The individual relation of each single physical activity and sedentary behavior (independent) and hypertension (dependent) was determined both with and without adjusting for covariates. Then, partition models were conducted with all physical behaviors simultaneously entered in the model to evaluate the relation between each behavior with hypertension. Two partition models and subsequent substitution models were calculated, one with total sedentary time, and the other distinguishing prolonged (>1 h) sedentary time. To estimate the effect of substituting activity and sedentary behaviors, total daily wear time and each activity behavior (i.e., sedentary behavior, standing, light physical activity and moderate‐to‐vigorous physical activity) were entered into the model simultaneously, except for the activity behavior to be substituted. See Model 1 and Model 2 for an example of replacing sedentary time.

**Model 1**. (Partition Model)

$$\begin{array}{c} \text{Hypertension}=\text{Intercept}+{ß}_{1}\text{Sedentary time}\\ \;+{ß}_{2}\text{Standing time}+{ß}_{3}\text{Light physical activity}\\ \;+{ß}_{4}\text{Moderate}-\text{to}-\text{vigorous physical activity}\\ \;+{ß}_{5}\text{Covariates}+\text{Error} \end{array}$$

**Model 2**. (Replacing sedentary time example)

$$\begin{array}{c} \text{Hypertension}=\text{Intercept}+{ß}_{1}\text{Standing time}\\ \;+{ß}_{2}\text{Light physical activity}+{ß}_{3}\text{Moderate}\\ \;-\text{to}-\text{vigorous physical activity}\\ \;+{ß}_{4}\text{Total wear time}+{ß}_{5}\text{Covariates}\\ \;+\text{Error}. \end{array}$$

Odds ratios (OR) and 95% confidence intervals (CI) were derived to assess the relation between activity substitutions on hypertension. ORs above 1 and below 1 indicated higher and lower risk of hypertension, respectively. If the CI of the OR included 1, the relation was considered non‐significant. An *α* = 0.05 was set to indicate statistical significance for all tests.

## 3 RESULTS

### 3.1 Participant demographics

Participant demographics are presented in Table 1. A greater proportion of females were White compared with males (*p* < 0.001), and females were more likely to have completed higher education (*p* < 0.001). Males were taller and weighed more than females (both *p* < 0.001). A greater proportion of males had hypertension compared to females (*p* < 0.001).

**TABLE 1:** Participant demographics by sex.

	Pooled (*n* = 4416)	Females (*n* = 2337)	Males (*n* = 2079)	*p*‐value
Height (cm)	169 ± 9	163 ± 6	177 ± 7	**<0.001**
Body mass (kg)	83.1 ± 18.2	77.1 ± 16.9	90.7 ± 16.9	**<0.001**
Diabetes	93 (2%)	37 (2%)	56 (3%)	**0.010**
Menopausal	–	112 (5%)	–	–
# Days	6.5 ± 0.9	6.5 ± 0.9	6.6 ± 0.9	**0.005**
Waking hours (h/day)	15.8 ± 1.2	15.7 ± 1.3	16.0 ± 1.2	**<0.001**
Sleeping hours (h/day)	8.1 ± 1.2	8.3 ± 1.2	8.0 ± 1.1	**<0.001**
Total PA (h/day)	1.9 ± 0.7	1.8 ± 0.7	1.9 ± 0.8	**<0.001**
LPA (h/day)	1.1 ± 0.4	1.1 ± 0.4	1.1 ± 0.5	**<0.001**
MVPA (h/day)	0.8 ± 0.4	0.8 ± 0.4	0.8 ± 0.4	0.863
Step count	8823 ± 3654	8652 ± 3504	9040 ± 3825	**<0.001**
Sedentary (h/day)	9.4 ± 2.0	9.1 ± 2.0	9.8 ± 2.0	**<0.001**
Prol Sed (h/day)	2.4 ± 1.5	2.4 ± 1.4	2.6 ± 1.6	**<0.001**
Standing (h/day)	4.5 ± 1.6	4.7 ± 1.6	4.3 ± 1.5	**<0.001**
STS transitions	54 ± 16	56 ± 16	53 ± 16	**<0.001**
Hypertension	1794 (24%)	756 (18%)	1038 (32%)	**<0.001**
Hypertension medication	127 (3%)	48 (2%)	79 (4%)	**<0.001**

*Note*: Sex comparisons completed using independent *t*‐tests or chi squared tests for continuous and categorical variables respectively (*α* = 0.05). Bold indicates *p* value for <0.05.

Abbreviations: LPA, light physical activity; MVPA, moderate to vigorous physical activity; PA, physical activity (i.e., stepping time); Prol Sed, prolonged (>1 h) sedentary time; STS, sit‐to‐stand transitions.

Participants wore the activPAL for 6.5 ± 0.9 (range: 4–8) days. Females wore the monitor for fewer hours than males (*p* < 0.001). Females exhibited less total sedentary time (*p* < 0.001) and less prolonged sedentary time (*p* < 0.001) compared to males. Females also accumulated less LPA (*p* < 0.001) and fewer steps/day (*p* < 0.001), but greater standing time (*p* < 0.001) and more sit‐to‐stand transitions (*p* < 0.001). There was no difference in MVPA between sexes (*p* = 0.863).

### 3.2 Single and partition models

Single‐activity and partition regression models are presented in Table 2. In the total sedentary partition model, LPA (OR = 0.93 [0.86, 1.00], *p* = 0.006) and MVPA (OR = 0.90 [0.83, 0.97], *p* = 0.006) were associated with lower odds of hypertension, whereas sedentary time (OR = 1.00 [0.98, 1.03], *p* = 0.838) and standing time (OR = 1.01 [0.98, 1.04], *p* = 0.384) were not. In the detailed sedentary breakdown model, only MVPA was associated with lower odds of hypertension, and all other variables (prolonged sedentary time, standing, and LPA) were not (all *p* > 0.054).

**TABLE 2:** Single variable models for entire sample, with accelerometer derived physical activity and postures predicting hypertension risk.

		OR	Low 95% CI	Upper 95% CI	*p*‐value
Single models					
Sed	Unadj	1.05	1.04	1.07	**<0.001**
	Adj	1.01	0.99	1.03	0.105
Prol Sed	Unadj	1.06	1.04	1.08	**<0.001**
	Adj	1.01	0.99	1.03	0.341
Stand	Unadj	0.96	0.95	0.98	**<0.001**
	Adj	0.99	0.98	1.01	0.468
LPA	Unadj	0.88	0.82	0.93	**<0.001**
	Adj	0.91	0.86	0.97	**0.006**
MVPA	Unadj	0.73	0.68	0.78	**<0.001**
	Adj	0.88	0.82	0.95	**<0.001**
Partition models					
*Total sedentary*					
Sed	Unadj	1.05	1.02	1.07	**<0.001**
	Adj	1.00	0.98	1.03	0.838
Standing	Unadj	1.02	0.99	1.05	0.181
	Adj	1.01	0.98	1.04	0.384
LPA	Unadj	1.04	0.96	1.12	0.337
	Adj	0.93	0.86	1.00	0.062
MVPA	Unadj	0.77	0.72	0.83	**<0.001**
	Adj	0.90	0.83	0.97	**0.006**
*Detailed sedentary breakdown*					
Prol Sed	Unadj	1.06	1.03	1.09	**<0.001**
	Adj	1.00	0.97	1.02	0.832
Standing	Unadj	1.02	0.99	1.05	0.169
	Adj	1.01	0.98	1.04	0.393
LPA	Unadj	1.04	0.97	1.13	0.263
	Adj	0.93	0.85	1.00	0.054
MVPA	Unadj	0.78	0.72	0.84	**<0.001**
	Adj	0.89	0.83	0.97	**0.005**

*Note*: Bold indicates *p* value for <0.05.

Abbreviations: CI, confidence interval; LPA, light‐physical‐activity; MVPA, moderate‐to‐vigorous physical activity; OR, odds ratio; sed, prolonged (>1 h) sedentary time; Sed, sedentary time; Stand, standing time.

In females, in both the total and detailed sedentary partition models, only standing time was associated with higher odds of hypertension (OR = 1.057 [1.017, 1.098], *p* = 0.005), whereas all other variables were not associated (all *p* > 0.198). In males, only MVPA was associated with lower odds of hypertension (OR = 0.863 [0.779, 0.955], *p* = 0.004) in both the total and detailed sedentary partition models. All other variables were not significant (all *p* > 0.135).

### 3.3 Substituting movement on stage 2 hypertension

**FIGURE 1:**
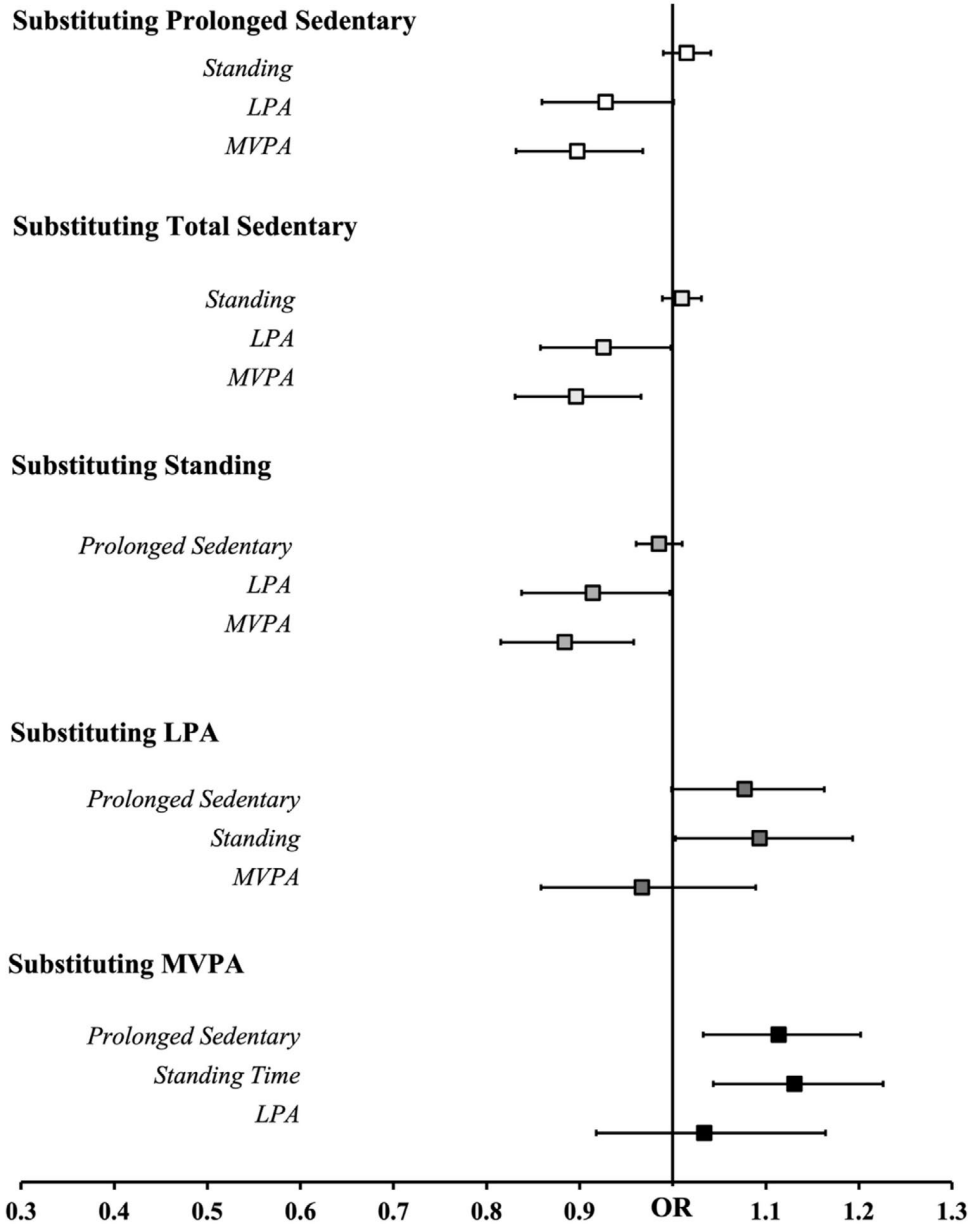
Forrest plot depicting isotemporal substitution adjusted for covariates (sex and body mass index) examining the impact of replacing 30 min of each physical activity/posture with another on hypertension. The odds ratios (OR ± 95% CI) represent the change in hypertension risk predicted by substituting one behavior with another. LPA, light‐intensity physical activity; MVPA, moderate‐to‐vigorous intensity physical activity. *p < 0.05. The vertical line is set at 1.

**FIGURE 2:**
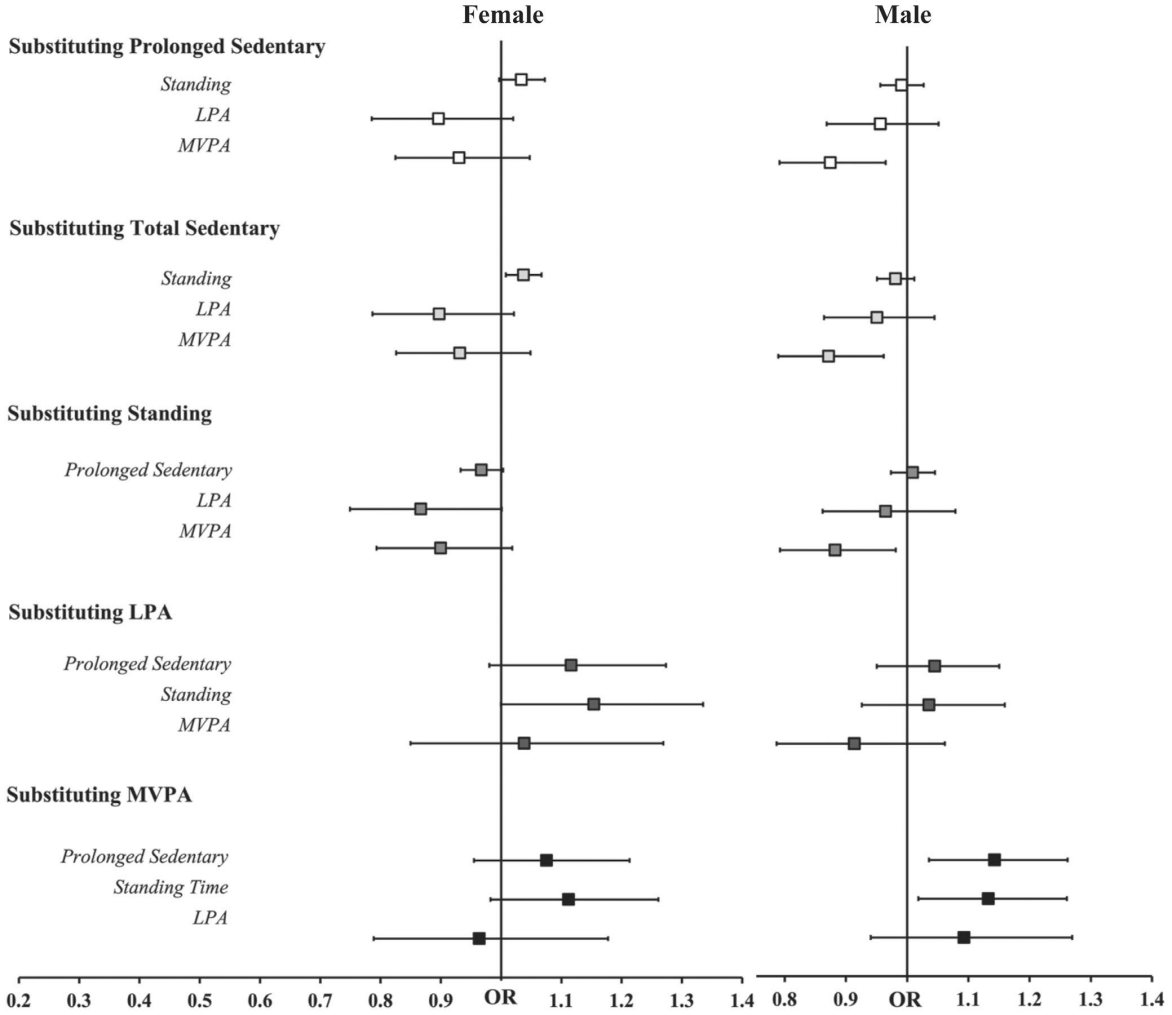
Forrest plot depicting sex‐stratified (Left = female, Right = male) isotemporal substitution adjusted for covariates (body mass index) examining the impact of replacing 30 min of each physical behavior with another on hypertension. The odds ratios (OR ± 95% CI) represent the change in hypertension risk predicted by substituting one behavior with another. LPA, light‐intensity physical activity; MVPA, moderate‐to‐vigorous intensity physical activity. *p < 0.05. The vertical line is set at 1.

Pooled sample substitution models are presented in Figures 1 and 2. Substituting 30 min total sedentary time with either LPA (OR = 0.926 [0.858, 0.998]) or MVPA (OR = 0.896 [0.831, 0.966]) was associated with lower odds of hypertension, whereas standing was not. Replacement of prolonged sedentary time with MVPA (OR = 0.898 [0.832, 0.968]) was associated with lower odds of hypertension, whereas substitutions of prolonged sedentary time with standing or LPA were not significant.

In the pooled sample, substituting LPA with standing (OR = 1.094 [1.003, 1.193]) was associated with higher odds of hypertension, while replacing LPA with MVPA was not. Lastly, replacing MVPA with prolonged sedentary time (OR = 1.114 [1.033, 1.202]) and standing (OR = 1.131 [1.043, 1.226]) was associated with higher odds of hypertension, whereas substituting MVPA with LPA was not.

In females, only replacing total sedentary time (OR = 1.04, [1.01, 1.07]) with standing was associated with higher odds of hypertension, but no other substitutions were significant (Figure 2). In males, replacing total sedentary time (OR = 0.872, [0.790, 0.962]), prolonged sedentary time (OR = 0.874, [0.792, 0.965]), or standing (OR = 0.882, [0.792, 0.981]) was associated with decreased odds of hypertension. No other substitutions were significant.

## 4 DISCUSSION

The purpose of the study was to investigate the impact of replacing habitual physical behaviors on hypertension, and to test the hypothesis that males exhibit a stronger association with physical behaviors and hypertension in a group of middle‐aged participants of the same age. Males exhibited a worse cardiovascular profile, were more sedentary, but had more stepping time than females. Overall, replacing sedentary time with any activity, and LPA with MVPA was associated with a lower incidence of hypertension. These observations were driven by males, with only two relations observed among females. Using an isotemporal substitution approach that controlled for impactful covariates, we demonstrate the potential favorable impact of lowering sedentary time and integrating MVPA on hypertension risk, particularly among males.

In the pooled sample, higher prolonged sedentary time was associated with increased incidence of hypertension, with prolonged activity being statistically similar to total sedentary time. These findings align with previous research suggesting that prolonged sitting is detrimental to cardiovascular health, even after accounting for physical activity levels (Warren et al., 2010). When sex‐stratified, the adverse effects of sedentariness were observed predominantly in males. Some, albeit conflicting, evidence suggests that males exhibit more pronounced impairments in blood pressure regulation and endothelial function following single bouts of prolonged sitting than females (Grgic et al., 2018). These sex differences may reflect underlying physiological variations in autonomic regulation and vascular responsiveness. Females generally demonstrate greater parasympathetic modulation, enhanced endothelial function, and reduced sympathetic vasoconstrictor dependence compared to males (Raberin et al., 2023), which may contribute to improved maintenance of vascular tone and blood pressure stability during periods of prolonged inactivity. Additionally, estrogen plays a central role in cardiovascular regulation by enhancing nitric oxide bioavailability, improving endothelial function, and attenuating sympathetic nervous system activity. Collectively, these mechanisms may mitigate the deleterious vascular consequences of sedentary behavior in females. We postulate that differences in the autonomic support of blood pressure between sexes, such as a less reliance on the sympathetic nervous system in females versus males and/or the cardioprotective nature of estrogen may be implicated in the attenuated dysfunction responses in females (Novella et al., 2012). With only a small proportion (5%) of the current study's female sample reporting being menopausal, as such, the relatively young, predominantly premenopausal female cohort examined here may represent a physiologically protected group. These findings support the presence of sex differences, suggesting that a sedentary lifestyle may have a more pronounced adverse impact on males during middle‐age. Given the substantially higher rates of hypertension among males during middle‐age, models that help males reduce sedentary time and engage in physical activity during these years may be paramount for addressing the development of cardiometabolic conditions with age. With that, a preventative lifestyle‐based model could still include females given the well‐established benefits of movement on other measures of health in middle‐aged females (Godoy‐Izquierdo et al., 2024). From an intervention perspective, reducing sedentary time may be a more practical and immediate goal than increasing MVPA which often demand greater effort and support (Keadle et al., 2017). Promoting frequent light activity and reducing long sedentary bouts may be more achievable, especially for individuals with limited mobility or time.

The study focused on middle‐aged individuals of the same age, a unique strength of the study. Results might differ if the population were older, as hypertension is more common in older adults and age‐related physiological changes could influence the relationship between physical activity and blood pressure (Warren et al., 2010). Studying middle‐aged individuals continues to offer significant value in understanding and preventing hypertension. This age group represents a critical window during which early signs of cardiovascular risk often begin to appear (Gray et al., 2011), allowing for timely interventions that may prevent more serious health outcomes later in life. Encouragingly, in the pooled sample with the greatest number of participants and thus statistical power, substituting sedentary or standing postures with LPA was associated with a positive effect. LPA has been shown to reduce cardiovascular risks (Raberin et al., 2023), and the results of our study support its efficacy in a relatively healthy group of middle‐aged adults. Easier‐to‐do, lower intensity activity may be efficacious for lowering hypertension risk. Standing time is often promoted in place of sedentary time and perceived as a superior physical activity, but the results of this study do not substantiate a major benefit for hypertension incidence in middle age, especially in females. Prior work using the UK Biobank also confirms that standing was not associated with cardiovascular disease risk but higher risk of orthostatic circulatory disease risk (Tudor‐Locke et al., 2019). While several questions remain as to the health benefits of standing (Novella et al., 2012), our results specific to hypertension in middle age support that models promoting movement in place of sedentary postures, whether light or MVPA, are beneficial, especially in males. Moreover, this cohort provides an important baseline for future longitudinal follow‐up, which may help clarify how sex‐specific associations between physical activity, sedentary behavior, and hypertension evolve across aging and menopausal transition.

This study's strengths include its large, population‐based sample who are all the same age and the inclusion of both males and females, enabling sex‐stratified analyses. It is important to consider that the female subgroup in our sample exhibited a lower prevalence of hypertension compared with males. This reduced prevalence may limit the statistical power to detect associations between behavioral or activity patterns and hypertension risk specifically in females. Consequently, some relations that are observable in males may be attenuated or undetectable in females due to this baseline difference. It may therefore be important to account for sex‐specific prevalence and consider hypertension status as a potential covariate when interpreting sex‐stratified analyses. Additionally, participants were not excluded based on cardiometabolic comorbidities, and adjustment was limited to BMI and sex‐stratified models. As the sample is intended to be population‐representative, individuals with conditions such as diabetes, dyslipidemia, or receiving hypertension treatment were included to preserve generalizability. However, these conditions may influence both physical activity behaviors and hypertension risk, and residual confounding cannot be excluded. Participants receiving treatment may have had variability in treatment adherence, duration of therapy, or level of blood pressure control that may have influenced the observed associations. The findings are inherently specific to middle‐aged adults, and the sample was predominantly white and relatively active individuals (~9000 steps/day). The activPAL device allowed for precise, objective measurement of physical behaviors (Unger et al., 2020; Writing Committee Members*, 2025). The use of a thigh‐worn monitor, compared to wrist or waist worn devices, offered more accurate postural data, highlighting the importance of precise device placement and standardization in cardiometabolic research. The study used a cross‐sectional design with accelerometer‐based measures and blood pressure outcomes, which is appropriate for identifying associations. To strengthen evidence of causality, longitudinal or interventional designs are warranted.

## 5 CONCLUSION

This study highlights that among middle‐aged adults from the 1970 British Cohort Study, replacing time in sedentary or standing postures with movement was associated with lower incidence of hypertension. Furthermore, MVPA was superior to LPA. These observations were more pronounced among males, who tended to be more sedentary and exhibit higher rates of hypertension than females. These findings underscore the importance of reducing time in sedentary postures as an individual‐level target for hypertension prevention, particularly in middle‐aged males.

## AUTHOR CONTRIBUTIONS

**Sophie E. Rayner:** Conceptualization; formal analysis; methodology. **Jolaine Katryne Bossé:** Formal analysis; investigation; methodology. **Corinne Brideau:** Formal analysis; investigation; methodology. **Jolaine Comeau:** Formal analysis; investigation; methodology. **Jessie Drouin:** Formal analysis; investigation; methodology. **Jalila Jbilou:** Methodology; supervision. **Myles W. O'Brien:** Conceptualization; methodology; project administration; supervision.

## CONFLICT OF INTEREST STATEMENT

The authors declare no conflicts of interest.

## ETHICS STATEMENT

Ethical approval for each wave of data collection in the 1970BCS was obtained by the CLS frm the appropriate ethics committees. Data are currently publicly openly available to researchers on their website.

## Data Availability

Data are available from the CLS Resource Centre.
